# Chlorella Growth Factor: Biochemical Identity, Redox-Active Mechanisms, Nutritional Relevance, and Translational Implications

**DOI:** 10.3390/nu18081250

**Published:** 2026-04-15

**Authors:** Wojciech Rzeski, Weronika Rzeska

**Affiliations:** 1 Department of Functional Anatomy and Cytobiology, Institute of Biological Sciences, Maria Curie-Skłodowska University, Akademicka 19, 20-033 Lublin, Poland; 2 Doctoral School of Medical Sciences, Medical University of Lublin, 20-093 Lublin, Poland; weronikarzeska@gmail.com

**Keywords:** Chlorella Growth Factor, bioactive nutrients, dietary nucleotides, oxidative stress, NAD metabolism, extracellular matrix remodeling, immunometabolism, immunonutrition, functional foods, nutrigenomics, CGF standardization, extraction methodology

## Abstract

Chlorella Growth Factor (CGF) is a nucleotide-rich, water-soluble intracellular fraction derived from disrupted Chlorella biomass that has historically been described as a “growth-promoting” extract but remains poorly defined at the molecular level. In this review, we propose that CGF should not be interpreted as a classical receptor-binding growth factor, but rather as a heterogeneous, nucleotide-dominant metabolic fraction that may modulate cellular redox balance and biosynthetic capacity. We integrate available evidence on CGF characterization, including A260-based analytical indices, mineral-dependent biosynthesis, and extraction methodologies, with mechanistic observations from in vitro, animal, and applied biological systems. Across these contexts, CGF-associated fractions have been reported to influence redox-sensitive pathways, including NAD(H)/NADP(H)-linked processes, MAPK/AP-1 signaling, extracellular matrix regulation, and humoral immune responses. However, most mechanistic evidence remains indirect, and compositional heterogeneity limits direct comparability across studies. From a nutritional perspective, CGF contributes minimal macronutrient value but may provide conditionally relevant dietary nucleotides, amino acids, and redox-active metabolites that support metabolic processes under stress conditions. Observed biological effects are consistent with a model of metabolic permissiveness, in which CGF-associated fractions may support endogenous cellular functions rather than directly initiating signaling cascades. Key translational challenges include the lack of compositional standardization, limited nucleotide speciation, variability in extraction protocols, and the absence of pharmacokinetic and controlled human studies using well-characterized CGF preparations. Overall, CGF may be conceptualized as a candidate dietary bioactive with redox-centered and metabolically permissive properties. Further work integrating standardized analytical frameworks with mechanistic and clinical validation will be required to establish its role in human nutrition and functional food applications.

## 1. Introduction

Microalgae have attracted increasing attention as sustainable sources of bioactive compounds for human nutrition and functional food development [1,2,3,4,5,6]. Among them, Chlorella species represent one of the most extensively studied genera, characterized by high protein content, essential amino acids, nucleotides, vitamins, and diverse antioxidant molecules [2,3,7]. Beyond their macronutrient profile, Chlorella-derived preparations have been investigated for potential immunomodulatory, metabolic, and antioxidant effects in experimental and clinical settings [8,9,10].

Chlorella Growth Factor (CGF) refers to a low-molecular-weight, water-soluble fraction obtained from disrupted *Chlorella* cells. Unlike whole biomass supplements, CGF is enriched in nucleotides, small peptides, amino acids, and redox-relevant metabolites. Although early investigations associated CGF with growth-supportive effects in vitro, its compositional heterogeneity and mechanistic underpinnings remain incompletely defined. Most clinical studies have evaluated whole Chlorella preparations rather than isolated CGF fractions, complicating translational interpretation [10].

Despite accumulating experimental observations, no comprehensive synthesis currently integrates CGF composition, analytical variability, molecular mechanisms, and translational limitations within a unified framework. The present review aims to (i) characterize CGF from an analytical perspective, (ii) summarize evidence regarding its redox- and metabolism-related biological effects, (iii) discuss potential relevance of CGF within stress-adaptive and redox-sensitive biological contexts, and (iv) critically discuss standardization and clinical interpretation challenges relevant to nutraceutical development. This review is narrative and integrative in nature and does not follow a formal systematic review protocol. Importantly, CGF should be interpreted as a heterogeneous, extraction-dependent set of fractions rather than a single chemically defined entity, which has implications for the interpretation of mechanistic and translational evidence.

## 2. Biochemical Identity, Production Variables, and Analytical Challenges

Chlorella Growth Factor (CGF) refers to a water-soluble intracellular fraction obtained from disrupted *Chlorella* biomass. Although historically described as a “growth factor”, CGF does not represent a single receptor-binding signaling molecule. Instead, it comprises a composite mixture of low-molecular-weight, metabolically active constituents, with a predominance of nucleotides and nucleotide-related compounds. An overview of the biochemical composition and functional interpretation of CGF is provided in Table 1 [11,12].

CGF has historically been defined operationally. The most widely used descriptor is the so-called CGF index, typically based on absorbance at 260 nm (A260), often expressed in an index form such as:

CGF index = A260 × dilution factor × extract mass (or volume)/biomass input

A structured overview of reported extraction parameters, CGF index definitions, mineral modulation (Mg^2+^/Fe^2+^), and analytical characterization methods is provided in Table 2.

This table summarizes reported extraction strategies, operational definitions, and analytical approaches used to characterize Chlorella Growth Factor (CGF) and related aqueous fractions. It highlights variability in methodology, including hot water extraction, enzymatic hydrolysis, and ultrasonic disruption. Analytical limitations of the A260-based CGF index and the need for LC–MS-based compositional standardization are emphasized. NAD(H)/NADP(H)-associated metabolites have been reported in selected metabolomic analyses, suggesting redox-relevant molecular signatures.

This approach assumes that the principal extractable components responsible for activity absorb strongly at 260 nm, consistent with nucleic acid-associated chromophores. Nevertheless, A260 is a coarse metric. It does not discriminate between intact RNA fragments, oligonucleotides, free nucleotides, nucleosides, or degradation products; nor does it exclude contributions from other UV-absorbing species. Moreover, extraction-dependent molecular weight distribution can shift A260 without proportionate changes in biological activity. For these reasons, CGF index should be interpreted as a process indicator rather than a compositional standard [12,13].

Extraction methodology is a dominant driver of CGF heterogeneity. Hot water extraction (commonly 90–100 °C) releases water-soluble intracellular molecules including nucleotide fragments, peptides, amino acids, and cofactors. Enzymatic pretreatment (proteases and/or carbohydrases) can markedly increase soluble fraction recovery and CGF index, likely by liberating nucleotide–peptide complexes from intracellular structures and partially hydrolyzing macromolecular assemblies into soluble bioactive fragments [13]. Ultrasonic-assisted extraction further modifies disruption efficiency and molecular size distribution, and may alter the relative abundance of bioactive constituents in the resulting extract, implying that extraction energy and time may influence biological potency [13].

Upstream cultivation variables add another layer of variability. Mineral availability (notably Mg^2+^ and Fe^2+^) modulates intracellular biosynthetic pathways and is reported to influence CGF yield and composition. Mg^2+^ is central to ribosomal function and nucleotide metabolism, while Fe^2+^ impacts redox enzymes and metabolic flux. Optimized Mg^2+^/Fe^2+^ concentrations have been associated with enhanced CGF formation, implying that intracellular metabolic state at harvest can strongly influence extract composition [11]. Beyond minerals, trophic mode (autotrophic/mixotrophic), light intensity, nitrogen status, temperature, and stress conditions likely alter intracellular nucleotide pools and redox-related metabolites—yet these parameters are rarely harmonized across studies [11,13].

Modern compositional profiling has begun to clarify CGF’s biochemical nature. LC–MS analyses detect multiple amino acids within CGF-associated fractions, including glutamate, alanine, tyrosine, tryptophan, phenylalanine, methionine, valine, leucine/isoleucine, and proline [12]. In some reports, nucleotide-associated content is described as a major proportion of extract mass, consistent with strong A260 absorbance. A particularly important recent direction is metabolomic comparison between Chlorella-derived extracts and fetal bovine serum (FBS), reporting overlap in metabolites related to NAD(H)/NADP(H) redox systems [14]. This does not imply that CGF “replaces serum” in a full functional sense, but it supports the hypothesis that CGF may provide redox-relevant substrates or cofactors contributing to biosynthetic and redox support capacity [11,15,16,17].

A major recurring pitfall in the field is conflation of whole-biomass and extract effects. Whole Chlorella contains proteins, lipids, pigments, cell wall polysaccharides, and fiber that independently influence antioxidant capacity, immune function, and lipid metabolism. CGF, in contrast, is the water-soluble intracellular fraction and is expected to be enriched in nucleotide- and small metabolite-associated components. Several animal studies suggest that CGF can modulate immune parameters (e.g., immunoglobulins) without markedly enhancing growth performance—an important signal that CGF is not simply a concentrated nutrition source but may exert more specific metabolic/immune actions [18].

Despite growing evidence, CGF remains analytically underdefined and should be interpreted as a family of extraction-dependent aqueous fractions rather than a single chemically defined entity, which limits direct comparability across studies [11,12,13]. Key gaps include the lack of quantitative nucleotide speciation, limited reporting of molecular weight distribution, absence of certified reference materials, inconsistent extraction protocols, and limited batch-to-batch characterization. All citations in this review have been critically verified against the content of the cited sources. Where references provide mechanistic context rather than direct empirical support, this is explicitly reflected in the surrounding text through appropriately hedged language (e.g., “consistent with”, “suggests”, “may support”).

Throughout this review, the following terms are used with distinct meanings: (i) “CGF” or “Chlorella Growth Factor” refers to a hot-water-soluble aqueous intracellular fraction operationally defined by UV absorbance (A260 index); (ii) “CGF-associated fractions” refers to the heterogeneous family of related extracts that meet CGF operational criteria but differ in composition depending on extraction method; (iii) “aqueous Chlorella extract” refers to water-soluble fractions from Chlorella that may or may not be characterized as CGF; and (iv) “Chlorella-derived fractions” is used where the source is Chlorella biomass but the fraction type (whole extract, peptide fraction, polysaccharide) is distinct from CGF. Evidence specifically derived from purified or well-characterized CGF preparations is distinguished from evidence derived from whole Chlorella biomass or other fractions throughout.

## 3. Molecular Signaling Context and Comparative Perspective

Chlorella Growth Factor occupies an unusual conceptual position within the landscape of biologically active compounds. Its historical designation as a “growth factor” contrasts sharply with the absence of a defined receptor-binding protein or peptide sequence analogous to canonical growth factors such as epidermal growth factor (EGF), fibroblast growth factor (FGF), platelet-derived growth factor (PDGF), or transforming growth factor-β (TGF-β). This semantic inconsistency has contributed to conceptual ambiguity in the literature. When placed within the broader framework of cellular signaling biology, CGF aligns more closely with metabolic and redox modulators than with ligand-driven receptor agonists [11,12].

Classical growth factors exert their biological effects through highly specific ligand–receptor interactions, typically activating receptor tyrosine kinases or serine/threonine kinase receptors that initiate phosphorylation cascades (PI3K/AKT, MAPK/ERK, JAK/STAT). These cascades are rapid, are dose-dependent in narrow concentration ranges, and exhibit clear receptor saturation kinetics. In contrast, reported CGF effects are often gradual, context-dependent, and most pronounced under stress or nutrient-limited conditions. Rather than overriding cellular programs through receptor activation, CGF appears to modulate the intracellular environment in ways that enable or facilitate endogenous signaling networks [19,20].

This distinction becomes particularly evident when examining fibroblast proliferation data. In serum-replete conditions, CGF does not appear to function as a primary mitogenic stimulus, and its proliferative effects may be limited compared with those of classical growth factors. CGF-associated fractions are more consistently reported to support cell proliferation under stress or serum-limited conditions, likely by reducing metabolic constraints rather than activating growth factor receptors. This pattern suggests that CGF does not act as a primary mitogenic trigger but rather reduces metabolic constraints that would otherwise limit cell cycle progression. Such a permissive effect aligns with increased nucleotide availability, improved redox buffering, and enhanced NAD(H)/NADP(H)-dependent enzymatic activity [12,14].

Comparison with exogenous nucleotide supplementation further clarifies this point. In clinical nutrition, nucleotides are considered conditionally essential during periods of rapid cell division, immune activation, or tissue repair. Supplementation has been shown to support intestinal and immune cell proliferation in stress states. CGF, being nucleotide-dominant in many preparations, may operate in a similar biochemical space, but with additional complexity. Unlike isolated nucleotides, CGF contains a heterogeneous mixture of nucleotide fragments, amino acids, and redox-relevant metabolites. This compositional diversity may enable synergistic interactions that influence multiple nodes of cellular metabolism simultaneously [21,22,23,24].

The relationship between CGF and NAD metabolism warrants particular attention. NAD^+^ serves not only as a redox cofactor but also as a substrate for sirtuins, PARPs, and CD38-mediated reactions. Age-associated decline in NAD^+^ availability has been implicated in mitochondrial dysfunction and impaired DNA repair. While direct quantification of specific NAD precursors within CGF remains limited, metabolomic overlap with NAD(H)/NADP(H)-associated species suggests functional convergence. CGF should not be equated with purified NAD precursors such as nicotinamide mononucleotide (NMN) or nicotinamide riboside (NR); however, the broader provision of nucleotide substrates and redox-active metabolites may indirectly support NAD-dependent processes [16,17,25,26]. CGF-associated metabolites may support NAD-dependent processes indirectly by providing nucleotide-related substrates and redox-relevant cofactors; however, direct evidence linking CGF to modulation of intracellular NAD^+^ pools remains limited.

When compared with other microalgal extracts, CGF’s distinguishing feature is its intracellular, water-soluble origin. Many Spirulina or Chlorella whole-biomass studies emphasize pigments (e.g., phycocyanin), polysaccharides, or lipid-derived bioactivity. CGF instead isolates the aqueous intracellular milieu, enriching for small metabolites and nucleotide-associated components. Consequently, its mechanistic profile aligns less with antioxidant scavenging and more with metabolic regulation [3,5,7].

This reframing also helps interpret cross-system observations. The capacity of CGF to enhance microbial fermentation efficiency [27,28], support fibroblast proliferation in serum-restricted media [12,14], and influence humoral immunity in animal models [18], together with the modulation of redox-sensitive ECM pathways observed with Chlorella-derived fractions [14,29], is consistent with a possible unifying framework: CGF-associated effects are consistent with a conceptual model of increased metabolic capacity of cells under stress conditions. That is, rather than instructing cells what to do, CGF may increase their capacity to do it.

Such a model is consistent with redox biology. Cellular signaling pathways are highly sensitive to redox state. Mild increases in NADPH availability can shift reactive oxygen species (ROS) signaling from destructive to regulatory [29]. Enhanced nucleotide pools can support DNA replication and repair. Improved redox buffering can attenuate stress-induced activation of AP-1 and NF-κB without abolishing necessary signaling. Within this framework, CGF emerges not as a discrete signaling molecule, but as a metabolic context modifier [16,17,30]. The terms “metabolic permissiveness” and “redox modulation” describe related but distinct aspects of CGF action. “Metabolic permissiveness” refers to the capacity of CGF-associated substrates (nucleotides, redox cofactors) to alleviate rate-limiting biosynthetic constraints, thereby enabling endogenous cellular programs to proceed. “Redox modulation” refers specifically to changes in NAD(H)/NADP(H) ratios, ROS signaling thresholds, or antioxidant enzyme capacity. Importantly, metabolic permissiveness does not imply direct activation of signaling pathways, but rather facilitation of existing cellular programs by alleviating metabolic and redox constraints. These concepts are used consistently in this sense throughout the review.

## 4. Redox-Dependent Regulation of Proliferation and Extracellular Matrix Homeostasis

The most consistent mechanistic theme emerging from modern CGF studies involves redox-sensitive regulation of cell function, particularly in fibroblast and ECM contexts. A conceptual reframing is required: rather than interpreting CGF as a direct mitogenic trigger, available evidence supports the view that CGF-associated fractions influence intracellular metabolic state and redox balance, thereby enabling biosynthetic activity under stress and shifting ECM turnover toward repair [14,29].

Central to this view is the NAD(H)/NADP(H) axis. NAD^+^/NADH regulates glycolysis, mitochondrial respiration, and DNA repair pathways, while NADP^+^/NADPH supplies reducing equivalents for antioxidant systems (glutathione, thioredoxin) and anabolic processes. Metabolomic overlap between Chlorella-derived extracts and FBS, including NAD(H)/NADP(H)-related metabolites, provides a biochemical basis for hypothesizing that CGF supports redox buffering and biosynthetic capacity [14]. However, direct quantitative evidence linking CGF to modulation of intracellular NAD pools remains limited and requires further investigation. In serum-limited culture systems, aqueous Chlorella fractions have been reported to support cell proliferation when combined with defined growth factors, consistent with a metabolic-support (permissiveness) model rather than ligand–receptor mimicry [22,23].

UV-induced photoaging models provide a mechanistically precise window into redox–ECM coupling. UV exposure increases ROS generation and activates MAPK pathways (ERK/JNK/p38), which converge on AP-1 (c-Fos/c-Jun), driving MMP-1 upregulation and collagen degradation. Simultaneously, UV suppresses TGF-β signaling, reducing procollagen synthesis and shifting balance toward ECM catabolism. Complementary studies using Chlorella-derived peptides in UVB models demonstrate reduced AP-1 activation, decreased CYR61 (a matricellular stress protein associated with remodeling and inflammation), increased TGF-β receptor II expression, and enhanced procollagen expression [29]. Although peptide fractions are not identical to CGF, the mechanistic overlap supports a broader model in which Chlorella-derived intracellular fractions modulate stress-responsive transcription programs controlling ECM turnover [29,31,32,33].

Wound repair is intrinsically redox-dependent: ROS act as signaling intermediates necessary for defense and repair, but excessive oxidative burden impairs fibroblast function and collagen synthesis. Although direct in vivo evidence for CGF-specific wound healing effects is currently lacking and remains an important limitation for translational interpretation, the redox and ECM mechanisms described above are consistent with a model in which CGF-associated fractions could support tissue repair capacity under oxidative stress conditions. This concept remains to be directly validated in controlled animal or human wound models.

Together, these findings are consistent with a model of an integrated mechanism in which CGF-associated fractions may enhance redox buffering capacity (potentially via NAD(H)/NADP(H)-related metabolite availability and/or stimulation of endogenous antioxidant systems), attenuate stress-induced overactivation of MAPK/AP-1 pathways, restore collagen synthesis programs (via reduced TGF-β suppression and improved transcriptional environment), and rebalance TIMP/MMP equilibrium toward ECM preservation [31,32,33].

Key in vitro findings regarding redox-sensitive signaling modulation, MAPK/AP-1 regulation, TIMP/MMP balance, and collagen gene regulation are summarized in Table 3 [31].

This table integrates mechanistic findings from in vitro and in vivo studies evaluating CGF or related aqueous fractions. Reported effects include modulation of TIMP/MMP balance, suppression of AP-1 signaling, enhancement of collagen gene expression, redox-sensitive transcriptional regulation, and immune activation. The table differentiates between purified CGF fractions and whole biomass interventions to clarify mechanistic interpretation.

## 5. Immunometabolic and Systemic Effects: Humoral Immunity, Oxidative Resilience, and Nutrigenomic Signaling

Systemic effects reported for CGF and related aqueous fractions align with the concept of immunometabolic coupling. Immune activation is metabolically expensive and redox-sensitive; conversely, metabolic disorders are characterized by chronic low-grade inflammation and oxidative stress. CGF-associated nucleotide-rich fractions may influence this interface by providing substrates for biosynthesis and supporting redox homeostasis during immune responses [18,22,23,34,35]. Where evidence derives from whole Chlorella preparations, attribution of effects specifically to CGF fractions should be interpreted with caution.

The evidence summarized in this section can be classified according to intervention type: (A) direct CGF evidence—studies using CGF preparations defined at the product or experimental level (e.g., An et al. [18]); (B) aqueous Chlorella extract evidence—studies using aqueous extracts functionally analogous to CGF but not formally characterized as such (e.g., Ng et al. [12]); and (C) whole Chlorella biomass evidence—studies or reviews involving whole-biomass supplements, in which effects cannot be attributed specifically to CGF fractions (e.g., Abdel-Tawwab et al. [34], Ahmad et al. [35]). Where whole-biomass evidence is used to support mechanistic hypotheses, this is explicitly indicated and interpreted with appropriate caution.

Animal studies show that CGF supplementation can increase serum immunoglobulin levels (IgG and IgM), in some cases without commensurate growth promotion [18]. This pattern is informative: it suggests that CGF does not function merely as concentrated nutrition, but may facilitate immune cell activation. A plausible biochemical rationale is nucleotide availability. Rapid proliferation of B cells and plasma cell differentiation require robust nucleotide synthesis for DNA replication and antibody gene transcription. Exogenous nucleotide-rich fractions could reduce metabolic bottlenecks during immune activation, particularly under stress or limited nutrient availability [18,22,23,34,35,36].

In aquaculture infection models, Chlorella supplementation improves survival during pathogen challenge and increases antioxidant enzyme activity and other immune-related biomarkers [34,35]. While many aquaculture studies use whole biomass, they provide valuable stress-system models: aquatic organisms in high pathogen and oxidative stress contexts reveal whether interventions improve systemic resilience. The combination of enhanced survival and improved antioxidant enzyme activity supports a redox–immune mechanism rather than a purely nutritive one [34,35].

Metabolic regulation is another recurring theme. Dietary CGF or Chlorella-derived interventions have been associated with changes in lipid parameters and fatty acid composition in animal systems. Redox state is tightly coupled to lipid metabolism: NAD^+^ availability regulates β-oxidation and mitochondrial function, while NADPH supports fatty acid synthesis and antioxidant defense. Thus, CGF-associated shifts in NAD(H)/NADP(H) balance and redox buffering could plausibly influence lipid metabolic fluxes [37].

Human translational evidence is increasingly supported by nutrigenomic studies. In individuals with metabolic risk factors, Chlorella supplementation has been reported to modulate gene expression patterns associated with insulin signaling and lipid metabolism, accompanied by improvements in metabolic biomarkers [38]. While such trials commonly involve whole Chlorella rather than purified CGF, aqueous fractions likely contribute to observed effects via metabolically relevant signaling and redox-related pathways. Transcriptomic shifts provide mechanistic plausibility beyond simple antioxidant scavenging, suggesting that Chlorella-derived interventions can influence regulatory networks [37,38].

Animal and translational observations including humoral immune modulation, oxidative stress resilience, and lipid-related metabolic changes are summarized in Table 4 [18,34,35].

This table summarizes systemic, metabolic, and translational observations from animal and human studies. Reported outcomes include immunoglobulin modulation, oxidative stress reduction, altered lipid metabolism, nutrigenomic changes, fermentation enhancement, and industrial biorefinery integration. The table emphasizes the emerging concept of redox–immune–metabolic coupling.

## 6. Nutritional Value and Dietary Relevance of Chlorella Growth Factor

From a nutritional science perspective, the relevance of Chlorella Growth Factor (CGF) should be interpreted within the framework of dietary bioactives rather than pharmacological agents. CGF is not a purified molecule but a water-soluble intracellular fraction derived from edible microalgal biomass, and its potential value lies in shaping the nutritional and metabolic milieu rather than in direct receptor-mediated signaling.

Unlike whole Chlorella biomass, CGF does not meaningfully contribute to macronutrient intake in terms of protein, lipid, or fiber supply. Its nutritional significance is therefore qualitative rather than caloric. CGF preparations are enriched in low-molecular-weight, water-soluble components, including nucleotide-associated material, nucleosides, free amino acids, and redox-relevant metabolites, which are typically underrepresented in conventional nutrient profiling.

Dietary nucleotides are increasingly recognized as conditionally essential nutrients, particularly during periods of rapid cell division, immune activation, tissue repair, or metabolic stress. Endogenous de novo nucleotide synthesis is energetically demanding and tightly coupled to cellular redox state. In such contexts, exogenous nucleotide availability can reduce metabolic bottlenecks and support anabolic processes. While quantitative nucleotide speciation remains incomplete for many CGF preparations, strong A260 absorbance and compositional profiling support enrichment in nucleotide-associated material [21,22,23,24].

A key consideration in evaluating CGF’s nutritional relevance is the bioavailability and pharmacokinetics of its nucleotide constituents. Dietary nucleotides are absorbed in the small intestine primarily as nucleosides and free bases following enzymatic hydrolysis by intestinal phosphatases and nucleosidases. Intestinal absorption capacity for exogenous nucleotides is finite and saturable; absorbed nucleotides are predominantly incorporated via salvage rather than de novo synthesis pathways, with the proportion absorbed depending on form, food matrix, and physiological state [21,22,23,24,39]. Quantitative bioavailability data for dietary nucleotides in humans remain limited and are not available for CGF-associated nucleotide fractions specifically. Absorbed nucleotides enter salvage pathways preferentially in rapidly dividing tissues (intestinal epithelium, lymphocytes), reducing the energetic cost of de novo synthesis. For CGF specifically, pharmacokinetic data are absent: no controlled studies have characterized nucleotide absorption, distribution, or urinary excretion following oral CGF administration. This constitutes a critical gap; without pharmacokinetic data, the dose-dependency of any biological effect cannot be established, and translational inferences from in vitro or animal models must be made with caution.

Amino acids detected in CGF-associated fractions may further act as metabolic co-substrates, supporting nitrogen handling and biosynthetic reactions. Although absolute quantities are typically modest compared with whole-biomass protein, their contribution may be nutritionally relevant as part of an integrated redox–metabolic support matrix.

Within human nutrition CGF is best evaluated as a functional dietary fraction positioned between traditional nutrients and pharmacological interventions. Its relevance is likely greatest in contexts characterized by increased biosynthetic demand or oxidative stress, where dietary nucleotides and metabolically active small molecules may support physiological resilience. Rigorous compositional characterization, bioavailability studies, and controlled human trials are required to determine whether mechanistic hypotheses translate into measurable health outcomes.

## 7. Translational, Biotechnological, and Regulatory Landscape

Moving CGF from experimental extract to validated functional ingredient requires addressing production, processing, safety, and regulatory classification. Mechanistic plausibility alone is insufficient; reproducibility and standardization are prerequisites for translation.

CGF has demonstrated utility beyond direct supplementation. In fermentation systems, aqueous Chlorella extracts have been reported to enhance microbial growth and modify metabolite profiles in plant-based fermentation, including changes in amino acid patterns and increased accumulation of bioactive metabolites [28]. This suggests that CGF can act as a metabolic enhancer across biological domains, consistent with nucleotide/redox permissiveness [28].

Industrial feasibility may be improved through biorefinery integration. Sequential extraction strategies allow recovery of CGF as a first aqueous fraction, followed by extraction of lipids and carotenoids from the residual biomass, improving process economics and reducing waste [13]. Nevertheless, such cascades introduce additional variability: thermal and enzymatic steps used for CGF extraction can alter downstream components and potentially affect CGF composition itself.

Sensory profile is a practical constraint. Aqueous Chlorella extracts often have strong odors from volatile compounds. Deodorization processes can improve acceptability, but may also remove small metabolites that contribute to bioactivity. Without pre/post deodorization compositional profiling, it is not possible to conclude whether biological activity is preserved. Therefore, sensory optimization should be coupled with LC–MS fingerprint monitoring and functional bioassays [40]. Direct, CGF-specific studies evaluating the impact of deodorization on small-molecule retention and bioactivity remain scarce. Therefore, evidence from broader algae biomass and extract deodorization literature is used here as a technology-analog to frame likely trade-offs between odor mitigation and preservation of low-molecular-weight constituents.

Claims framed as “detoxification” should be avoided; the more defensible interpretation is modulation of metal bioavailability or accumulation observed in experimental models, which requires controlled human validation. Given the capacity of microalgae to bioaccumulate contaminants depending on cultivation conditions, routine batch testing (e.g., ICP-MS) and compliance with applicable maximum-level regulations are essential.

Regulatory classification differs between whole biomass and purified extracts. Whole Chlorella is widely consumed and often recognized as safe, whereas purified CGF lacks harmonized regulatory definition. Regulatory pathways typically require precise compositional description, manufacturing consistency, stability, safety, and (for claims) human efficacy data. Without compositional standardization, CGF remains difficult to regulate as a defined ingredient [39,41].

## 8. Knowledge Gaps and Research Priorities

Despite rapidly increasing mechanistic evidence, CGF research is constrained by several gaps:

Compositional ambiguity: reliance on A260-based CGF index rather than quantitative nucleotide speciation.

Methodological variability: inconsistent extraction protocols (temperature, enzymes, ultrasonication) and limited reporting of upstream culture parameters.

Incomplete molecular profiling: limited reporting of molecular weight distribution, peptide/nucleotide size ranges, and metabolite stability after processing.

Reference standards: lack of certified reference materials and standardized LC–MS fingerprints.

Lack of pharmacokinetic data: absence of human studies on absorption, distribution, and excretion of CGF-associated nucleotides.

Limited dose–response evidence: scarcity of controlled studies defining effective intake ranges.

Long-term safety: insufficient data on chronic exposure and safety in humans.

Regulatory ambiguity: lack of harmonized classification and compositional standards for CGF as a distinct ingredient.

Priority actions should include standardized extraction guidelines, LC–MS fingerprint development, quantitative nucleotide panels, redox flux studies, and controlled human trials with purified CGF.

These limitations and proposed priority actions are summarized in Table 5.

This table outlines critical limitations in current CGF research, including lack of compositional harmonization, insufficient nucleotide speciation, absence of pharmacokinetic studies, and limited human clinical trials. Priority actions are proposed to enable mechanistic validation, translational development, and regulatory harmonization.

## 9. CGF in the Context of Aging-Associated Molecular Pathways

Although CGF-associated redox and nucleotide mechanisms share conceptual overlap with aging-related pathways, direct evidence in aged biological systems remains scarce. Nevertheless, caution is required. Most CGF evidence derives from acute stress models or non-aged organisms. The extrapolation to intrinsic aging must be considered hypothetical. Definitive conclusions require experiments in aged animal models assessing NAD levels, sirtuin activity, mitochondrial respiration, senescence-associated secretory phenotype (SASP) markers, ECM stiffness, and functional outcomes [25,26,42].

Within a rigorous scientific framework, CGF should be regarded as a candidate modulator of stress-adaptive capacity within aging-related pathways rather than an established anti-aging intervention.

## 10. General Conclusions and Conceptual Reframing

The body of evidence synthesized in this review supports a substantive conceptual reframing of Chlorella Growth Factor. Although historically described as a “growth-promoting” extract, CGF does not conform to the defining characteristics of classical growth factors, which exert their effects through discrete ligand–receptor interactions and tightly regulated mitogenic signaling cascades. Instead, CGF is more accurately understood as a water-soluble, nucleotide-dominant intracellular fraction derived from an edible microalgal source, whose biological activity emerges from its capacity to modulate metabolic and redox context rather than to directly instruct cellular fate.

This reframing resolves several longstanding ambiguities in the literature. First, it explains why CGF-associated effects are observed across diverse biological systems including serum-limited mammalian cell cultures, fibroblast photoaging models, microbial fermentation, aquaculture infection challenges, and immune modulation without evidence of receptor saturation kinetics or narrow dose–response windows. Second, it aligns the operational definition of CGF, typically based on A260 absorbance, with enrichment in nucleotide-associated material rather than with proteinaceous growth factors. Third, it provides a coherent biochemical rationale for reported overlaps between Chlorella-derived aqueous extracts and NAD(H)/NADP(H)-related metabolic signatures.

From a nutritional science perspective, CGF should not be evaluated on the basis of macronutrient contribution or caloric density. Its potential value lies instead in the provision of dietary nucleotide-associated material, amino acids, and redox-relevant metabolites, which may become conditionally important during periods of increased biosynthetic demand, oxidative stress, immune activation, or tissue repair. CGF aligns with emerging concepts in functional nutrition, in which bioactive food-derived fractions influence metabolic resilience and adaptive capacity rather than serving solely as sources of energy or structural nutrients.

Mechanistically, the most consistent and experimentally supported effects of CGF-associated fractions converge on redox-sensitive regulatory pathways. In vitro and in vivo studies indicate modulation of MAPK/AP-1 signaling, restoration of TIMP/MMP balance, enhancement of collagen biosynthesis under oxidative stress, and attenuation of maladaptive extracellular matrix remodeling. These effects are most parsimoniously interpreted through a model of metabolic permissiveness, in which improved redox buffering and nucleotide availability enable endogenous repair and biosynthetic programs rather than replacing physiological signaling with exogenous stimuli.

At the systemic level, reported increases in humoral immune markers, improved resilience in infection and stress models, and nutrigenomic shifts in metabolic gene expression further support the hypothesis that CGF contributes to immunometabolic coupling. The observed dissociation between immune modulation and overt growth promotion in several animal studies reinforces the view that CGF does not function as a concentrated nutritional supplement, but rather as a modulator of metabolic capacity during immune and oxidative challenges.

CGF must be clearly distinguished from whole Chlorella biomass in both scientific interpretation and regulatory positioning. Whole-biomass consumption delivers a complex mixture of proteins, lipids, pigments, polysaccharides, and fiber, whereas CGF selectively isolates the aqueous intracellular fraction. Extrapolation from whole-biomass studies to CGF without compositional resolution risks conflating fundamentally different nutritional and biological mechanisms. Consequently, CGF should be treated as a refined functional fraction, whose efficacy and safety depend on extraction methodology, compositional consistency, dose, and food matrix interactions.

Despite its mechanistic plausibility and growing translational interest, CGF remains analytically underdefined. Critical gaps include the absence of quantitative nucleotide speciation, limited molecular weight distribution data, lack of certified reference materials, insufficient pharmacokinetic information, and a scarcity of controlled human trials using purified and well-characterized CGF preparations. These limitations preclude definitive conclusions regarding clinical efficacy and necessitate caution against premature therapeutic or anti-aging claims.

CGF should presently be regarded as a candidate dietary bioactive with redox-centered and metabolically permissive properties, rather than as a validated intervention. Future research priorities should focus on harmonized extraction and analytical standards, LC–MS-based compositional fingerprinting, targeted investigation of nucleotide and redox fluxes, and rigorously designed human intervention studies. Addressing these challenges will be essential for determining whether the mechanistic promise of CGF can be translated into reproducible, evidence-based applications within human nutrition and functional food science. At present, CGF should not be considered a clinically validated intervention.

**Figure 1:**
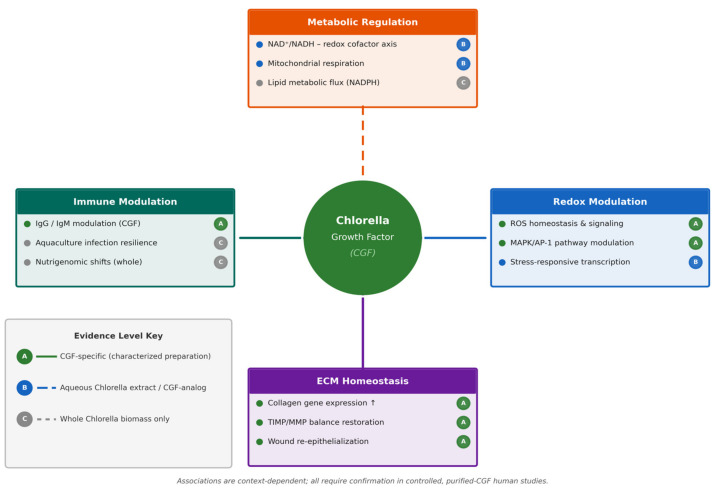
Integrative overview of the biological domains associated with Chlorella Growth Factor (CGF). This schematic presents CGF as a nucleotide-enriched, water-soluble intracellular fraction derived from edible microalgal biomass, positioned as a central conceptual node within interconnected biological domains. Surrounding modules illustrate major functional contexts in which CGF-associated fractions have been investigated, including metabolic regulation (NAD^+^-associated pathways, mitochondrial function, and lipid-related signaling), redox modulation (ROS homeostasis and signaling and stress-responsive transcriptional pathways), extracellular matrix (ECM) homeostasis (collagen gene expression and TIMP/MMP balance), and immune modulation (adaptive immune support and immunoglobulin regulation). Connecting lines indicate context-dependent modulatory associations rather than direct receptor-mediated signaling. Evidence levels are indicated by line style: solid lines represent effects documented in studies using characterized CGF preparations, whereas dashed lines indicate effects inferred from aqueous Chlorella extract or whole-biomass studies. All associations are context-dependent and remain to be confirmed in dose-controlled, purified-CGF human studies.

The integrative biological framework discussed throughout this review is summarized in Figure 1.

## Figures and Tables

**Table 1:** Biochemical composition and functional interpretation of Chlorella Growth Factor (CGF).

Component Class	Representative Constituents	Functional Relevance	Interpretative Remarks
Nucleotides and nucleosides	AMP, GMP, CMP, UMP; adenosine, guanosine; short RNA fragments	Support of DNA/RNA synthesis, cellular proliferation, and metabolic recovery	Dominant fraction of CGF; biological activity arises from substrate availability rather than receptor-mediated signaling
Oligonucleotides	Low-molecular-weight RNA-derived fragments	Enhancement of anabolic capacity and stress adaptation	Contribute to the metabolic permissive effects historically attributed to CGF
Free amino acids and short peptides	Glutamate, alanine, glycine; small peptides	Provision of anabolic substrates for protein synthesis and cellular maintenance	Nutritional and trophic role; not classical bioactive signaling peptides
B-group vitamins	Thiamine (B1), riboflavin (B2), pyridoxine (B6); strain-dependent cobalamin (B12)	Support of nucleotide metabolism, redox balance, and energy pathways	Composition varies with strain selection and cultivation conditions
Minerals and trace elements	Mg^2+^, Zn^2+^, Fe^2+^/Fe^3+^; trace Mn, Cu	Enzymatic cofactors for nucleic acid metabolism and redox regulation	Mineral availability during cultivation modulates CGF biosynthesis and composition

**Table 2:** Extraction Methods, Operational Definitions, and Analytical Characterization of CGF.

Study/Context	Biomass Source	Extraction Method	Analytical Definition	Key Molecular Findings	Major Limitations
Classical CGF production	*Chlorella* spp.	Hot water extraction (90–100 °C), centrifugation	A260-based CGF index	High UV absorbance suggests nucleotide-associated material	A260 does not distinguish RNA, nucleotides, or other chromophores
Enzymatic extraction studies	*C. vulgaris*	Protease and/or carbohydrase-assisted hydrolysis	Increased CGF index (up to >200%)	Higher soluble fraction recovery; release of nucleotide–peptide complexes	Variable enzyme cocktails; limited compositional quantification
Ultrasonic-assisted CGF fractions (e.g., CGF-3)	*C. pyrenoidosa*	Ultrasonication + hydrolysis	A260 measurement + bioassay	Enhanced collagen gene expression; increased fibroblast proliferation	Molecular size distribution rarely reported
Mineral optimization studies	*C. vulgaris*	Controlled Mg^2+^/Fe^2+^ culture conditions	CGF yield relative to biomass	Mg^2+^/Fe^2+^ enhance intracellular biosynthesis affecting extract composition	Lack of downstream molecular fingerprint validation
LC-MS metabolomic profiling	Aqueous Chlorella extract	Hot water extraction + LC-MS	Metabolomic fingerprint	Amino acids identified; NAD(H)/NADP(H)-related metabolites detected	Quantitative nucleotide speciation incomplete
Deodorization-processed CGF	Industrial CGF solution	Adsorption/thermal/solvent methods	Sensory + limited chemical analysis	Improved odor profile	Potential loss of small bioactive metabolites not systematically assessed

Potential loss of small bioactive metabolites not systematically assessed.

**Table 3:** Mechanistic Effects of CGF and Chlorella-Derived Fractions in Cellular and Animal Models.

Model System	Intervention Type	Key Molecular Targets	Observed Effects	Mechanistic Interpretation
Human skin fibroblasts (UVA)	CGF aqueous fraction	↑ TIMP-1, ↓ MMP-1, ↑ COL-I	Restoration of collagen synthesis; reduced ECM degradation	Redox-dependent modulation of MAPK/AP-1 signaling
Fibroblasts (UVB)	Chlorella-derived peptide (CDP)	↓ c-Fos/c-Jun, ↓ CYR61, ↑ TGF-βRII	Reduced photoaging markers	Suppression of AP-1 pathway; restoration of TGF-β signaling
Serum-free fibroblast culture	Aqueous Chlorella extract	NAD(H)/NADP(H)-related metabolites	Supported proliferation under low-serum conditions	Metabolic permissiveness via redox cofactor support
Shrimp infection model	Dietary Chlorella	Antioxidant enzymes, survival rate	Increased resistance to bacterial infection	Redox–immune coupling; enhanced oxidative resilience
Broiler immune model	CGF supplementation	↑ IgG, ↑ IgM	Enhanced humoral immune response	Nucleotide availability facilitating lymphocyte proliferation

**Table 4:** Immunometabolic and Translational Evidence Associated with CGF and Aqueous Chlorella Fractions.

Context	Population/Model	Primary Outcomes	Secondary Molecular Indicators	Translational Implications
Human metabolic risk study	Adults with high-risk metabolic markers	Improved lipid profile; altered insulin-related gene expression	Transcriptomic shifts in metabolic pathways	Potential immunometabolic modulation
Aquaculture stress model	Fish/shrimp under pathogen exposure	Increased survival; improved feed efficiency	Elevated antioxidant enzyme activity	Systemic oxidative resilience
Fermentation enhancement	Plant-based fermentation systems	Increased bioactive compound yield	Altered amino acid and metabolite profile	CGF as metabolic enhancer
Biorefinery cascade	Industrial microalgae processing	Sequential recovery of CGF + lipids/carotenoids	Process efficiency improvement	Economic feasibility
Deodorized CGF products	Functional food development	Improved sensory acceptability	Limited molecular characterization	Need for compositional preservation validation
Metal-handling studies	Animal models	Increased metal excretion	Possible chelation or absorption modulation	Requires human validation

**Table 5:** Knowledge Gaps, Methodological Limitations, and Research Priorities in CGF Investigation.

Domain	Current Limitation	Scientific Consequence	Priority Action	Translational Impact
Compositional definition	CGF defined primarily by A260 index	Molecular heterogeneity across studies	LC-MS-based compositional fingerprinting	Improved reproducibility
Nucleotide speciation	Lack of quantitative profiling of specific nucleotides	Unclear contribution of NAD precursors	Targeted metabolomics and nucleotide quantification	Mechanistic clarity
Molecular weight distribution	Absence of fractionation data	Unknown bioactive molecular range	Size-exclusion chromatography and proteomic mapping	Standardized product characterization
Extraction harmonization	Variable hot water/enzymatic protocols	Inconsistent CGF index values	Standardized extraction guidelines	Batch-to-batch consistency
Pharmacokinetics	No human absorption or distribution data	Uncertain systemic bioavailability	Controlled PK studies	Clinical translation
Dose–response	Limited human dose-ranging trials	Undefined therapeutic window	Randomized controlled trials	Evidence-based recommendations
Redox mechanism validation	Hypothesis-driven but indirect evidence	Incomplete causal mapping	NAD flux tracing and redox biosensor studies	Mechanistic validation
Long-term safety	Sparse chronic exposure data	Regulatory uncertainty	Long-term toxicity and safety studies	Regulatory approval pathway
Regulatory classification	No harmonized CGF category	Market ambiguity	Defined compositional specification	Nutraceutical positioning

## Data Availability

No new data were created or analyzed in this study. Data sharing is not applicable to this article.
